# Incorporation of α-Tocopherol into Pea Protein Edible Film Using pH-Shifting and Nanoemulsion Treatments: Enhancing Its Antioxidant Activity without Negative Impacts on Mechanical Properties

**DOI:** 10.3390/foods12102022

**Published:** 2023-05-17

**Authors:** Jingjing Cheng, Jing Wang, Leqi Cui

**Affiliations:** Department of Nutrition and Integrative Physiology, Florida State University, Tallahassee, FL 32306, USA; jc21bn@fsu.edu (J.C.); jw21b@fsu.edu (J.W.)

**Keywords:** pea protein, pH-shifting, emulsion, antioxidant, active film, elongation at break

## Abstract

The aim of this study is to develop an antioxidant film based on pea protein isolate (PPI) without sacrificing the packaging properties. To achieve this, α-tocopherol was incorporated to impart antioxidant activity to the film. We investigated the effects on film properties resulting from the addition of α-tocopherol in a nanoemulsion form and pH-shifting treatment of PPI. The results revealed that direct addition of α-tocopherol into un-treated PPI film disrupted film structure and formed a discontinuous film with rough surface, and thereby significantly decreasing the tensile strength and elongation at break. However, pH-shifting treatment in combination with the α-tocopherol nanoemulsion, formed a smooth and compact film, which greatly improved the mechanical properties. It also significantly changed the color and opacity of PPI film, but had little effects on film solubility, moisture content, and water vapor permeability. After the addition of α-tocopherol, the DPPH scavenging ability of PPI film was greatly improved and the release of α-tocopherol was mainly within the first 6 h. Additionally, pH-shifting and nanoemulsion did not affect the film’s antioxidant activity nor the release rate. In conclusion, pH-shifting combined with nanoemulsion is an effective method to incorporate hydrophobic compounds such as α-tocopherol into protein-based edible films without negative impacts on film mechanical properties.

## 1. Introduction

Pea protein has gained significant attention in the food industry due to its nutritional value, hypoallergenicity, and cost-effectiveness [1]. In addition to its nutritional benefits, pea protein can also be used to fabricate edible film, which is an innovative and sustainable alternative to synthetic materials that are commonly used in the food packaging industry. As consumers become increasingly aware of the environmental impact of conventional packaging materials, the demand for eco-friendly and bio-based packaging materials continues to grow, making pea protein a promising option for food researchers and manufacturers. In this context, research on edible films based on pea protein is ongoing and continuously making progress. For instance, Choi & Han [2] found that heat treatment at 90 °C over 5 min increased the mechanical properties of pea protein-based films. Later, Jia et al. [3] applied electrospinning technology to fabricate pea protein-pullulan nanofiber films and stated the surface hydrophobicity of crosslinked nanofiber films were significantly improved. Recently, our group reported that physical methods, such as ultrasound and high-pressure homogenization, and chemical methods, such as glycation, can greatly improve mechanical and water-resistance properties of pea protein film [4,5,6]. These studies have laid a solid foundation for the application of pea protein film in the food packaging industry.

Moreover, a good packaging material not only requires excellent packaging performance but also needs to play a role in preventing food spoilage and extending food shelf life. This is not only a consumer demand but also a business imperative for food companies. To achieve this, researchers have explored the use of α-tocopherol, a common food-grade natural antioxidant, as an additive in various edible films to enhance their antioxidant capacity [7,8,9,10]. Also, practical applications have shown that edible films containing α-tocopherol can slow down the oxidation rate of soybean oil and prolong the shelf life of peach [9,11]. However, it is important to note that in these studies [7,8,9,10], the improvement of antioxidant capacity was achieved at the expense of the mechanical properties of the edible films. This is because α-tocopherol is a hydrophobic compound that does not dissolve in water-based film-forming dispersions, which can result in poor dispersion and uneven distribution of α-tocopherol in the film matrix. Moreover, α-tocopherol may interact with other film components, such as proteins or polysaccharides, thus, affecting the physical and mechanical properties of edible films [12]. Lastly, it is well known that incorporating hydrophobic compounds into oil-in-water emulsion can greatly increase their solubility in aqueous solutions [13], so it is expected that addition of α-tocopherol in the form of a nanoemulsion would mix well into the film-forming solution with minimal impacts on film mechanical properties. However, a previous study suggested this may not be the case—even when α-tocopherol was added in the form of nanoemulsion, it could not prevent the decrease in film tensile strength [8].

The process of pH-shifting is a chemical method to modify protein structure. It involves adjusting the pH of a protein solution to either highly acidic or alkaline conditions, causing strong repulsion between protein particles and partial unfolding of the protein structure. After the protein has been partially unfolded, the pH is then adjusted back to neutral condition, allowing the partially unfolded protein particles to refold [14]. This results in a more flexible structure known as the molten globule state, which can improve protein interfacial adsorption behavior [15]. In fact, several studies have demonstrated that pH-shifting treatment can enhance the functional properties of proteins, such as solubility and emulsification [16,17]. However, there have been few studies focusing on the effects of pH-shifting on properties of protein-based film as well as its interaction with α-tocopherol. Given that pH-shifting can improve the interfacial properties of proteins, we propose that pH-shifting treatment could also aid in the incorporation of hydrophobic substances into protein-based films.

Based on the background above, this study aims to develop a PPI film with antioxidant capacity without sacrificing the film’s mechanical properties. The hypothesis is that pH-shifting treatment of PPI combined with addition of α-tocopherol in the nanoemulsion form enhances film antioxidant activity without negative impacts on the film properties. To test this, we evaluated the solubility, moisture content (MC), water vapor permeability (WVP), mechanical, and optical properties of the films. Also, antioxidant activity of PPI film was determined by measuring α-tocopherol release and DPPH radical scavenging activity.

## 2. Materials and Methods

### 2.1. Materials

Pea protein isolate (80% protein, 7% moisture, 6% lipid, 4% ash and 3% carbohydrates) was provided by Roquette America Inc. (Geneva, IL, USA). Analytical or higher grade glycerol, α-tocopherol, ethanol, and 2,2-diphenyl-1-picrylhydrazyl (DPPH) were purchased from Sigma (St. Louis, MO, USA). Medium chain triglycerides (MCT) were donated by IOI Oleo GmbH (MIGLYOL^®^ 812 N, Herrengraben, Hamburg, Germany). Ultrapure water (Barnstead Nanopure ultrapure water system, Thermo Scientific, Waltham, MA, USA) was used in all experiments.

### 2.2. pH-Shifting Treatment

To modify the PPI structure, pH-shifting treatment was applied to PPI dispersions following the method described previously [15], with modifications. Briefly, PPI powder was dispersed into water and stirred at room temperature for 4 h. Then, the dispersion was adjusted to pH 12.0 using 2 M NaOH and held at pH 12.0 for 1 h to induce unfolding. Following this step, PPI dispersion was titrated back to pH 7.0 using 2 N HCl to allow refolding. The pH-shifting-treated samples were freshly prepared for the following preparation of film-forming dispersions (Section 2.4).

### 2.3. Preparation of α-Tocopherol Nanoemulsion

The preparation of α-tocopherol nanoemulsion was referred to in a previous study [18] with some modifications. The oil phase contained 1 g of α-tocopherol dissolved in 1 g of MCT. The aqueous phase contained 1 g of PPI dispersed in 57 g of water. The coarse emulsion was prepared by mixing 2 g oil phase and 58 g aqueous phase using a high-speed homogenizer (IKA T25 digital ultra-turrax, Staufen, Germany) operated at 10,000 rpm for 3 min with a 10 s interval after each minute. The resulting coarse emulsion was then transformed into a nanoemulsion by passing it twice through a high-pressure homogenizer (Nano DeBEE high pressure homogenizer, BEE International, San Diego, CA, USA), operating at 15,000 psi. The α-tocopherol concentration in the final nanoemulsion was 1.67%. The emulsion particle size was measured by a Zetasizer Nano ZS (Nano ZS90, Malvern Panalytical, Malvern, UK) to be 280.90 ± 3.00 nm, so it was considered as nanoemulsion [19]. The nanoemulsion was freshly prepared for the following preparation of film-forming dispersions.

### 2.4. Preparation of Film-Forming Dispersions

The formulation was listed in Table 1. All final samples contained the same amount of PPI (7.5%), α-tocopherol (0.5%), and glycerol (3.75%) in the final film-forming dispersions. T0 is the PPI film without α-tocopherol; T1 (PPI+TOC) film is the film formed by direct addition of α-tocopherol (in ethanol) into PPI dispersion; T2 (PPI_pH12_+TOC) film is the film formed by treating PPI dispersion with the pH-shifting process, followed by direct addition of α-tocopherol (in ethanol); and T3 (PPI_pH12_+NanoTOC) film is the film formed by treating PPI dispersion with pH-shifting process, followed by addition of α-tocopherol nanoemulsion. Specifically, the PPI dispersion was prepared by adding PPI powder to distilled water and stirring the mixture for 4 h. Then, α-tocopherol dissolved in ethanol was added to PPI dispersions, followed by stirring for 1 h to prepare the T1 sample. The preparation steps for T2 and T3 samples were the same as for T1 except that the pH-shifting treated PPI dispersion was used for T2, and α-tocopherol nanoemulsion was added to the pH-shifting treated PPI dispersion for T3. After that, glycerol was added to the dispersions and stirred for 30 min. The samples were then subjected to vacuum for 30 min. Then, 8 g of the dispersions were poured on petri dish with a diameter of 9 cm. After drying for 2 days at room temperature, the films were peeled off and stored in a desiccator at 55% relative humidity for at least 48 h before analysis.

### 2.5. Characterization of PPI Films Loaded with α-Tocopherol

#### 2.5.1. Scanning Electron Microscopy (SEM)

Films were cut into 1 × 1 cm pieces and coated with uranium using a Cressington Carbon Coater (Cressington Scientific Intruments Ltd., Watford, UK). Surface morphology of films was observed using a field emission scanning electron microscope (FE-SEM) (FEI Nova 400 nanoSEM, FEI Company, Hillsboro, OR, USA). All micrographs were captured at an accelerating voltage of 5 kV and a magnification of ×2000.

#### 2.5.2. Solubility and Moisture Content (MC)

The solubility and MC of films were measured using the method of Pérez Córdoba & Sobral [20] with some modifications. Specifically, films were cut into pieces and placed in oven at 105 °C for 24 h after precise weight measurement. After that, the film pieces were weighed again and the percentage of weight loss of each film piece was defined as its MC. To measure solubility, the dried film pieces were immersed into 30 mL of distilled water for 24 h at room temperature. Then, the insoluble matter of film pieces was filtered, and weighed after completely drying using an oven at 105 °C. The solubility of film was calculated using following equation:

(1) $$\mathrm{Solubility}=\frac{W1-W2}{W1}\;\times \;100,$$

where W1 is the total weight of film pieces based on dry basis (g) and W2 is the weight of insoluble matter of film pieces based on dry basis (g).

#### 2.5.3. Water Vapor Permeability (WVP)

The WVP of films was measured according to the method described by ASTM Standard Test Method [21]. Each film (2 cm × 2 cm) was sealed on the top of a glass cup that was pre-filled with silica gel. Then, the glass cups were placed in a chamber containing distilled water. The weight changes of the cups were recorded periodically: every 8 h for the first day and then at 12-h intervals for the next two days. WVP was calculated using following equations:

(2) $$\mathrm{WVT}=\frac{\Delta m}{\Delta t\;\times \;A}$$

(3) $$\mathrm{WVP}=\frac{\mathrm{WVT}\;\times \;l}{\Delta p}$$

where WVT is water vapor transmission rate; Δm/Δt is the slope of linear regression equation of weight gain (g) as a function of time (h); A is the area of film exposed to the environment (m^2^); l is the film thickness (mm); and Δp is the vapor pressure difference through the film (2.895 kPa).

#### 2.5.4. Tensile Strength (TS) and Elongation at Break (EAB)

TA.XTplus texture analyzer (Stable Micro System Ltd., Godalming, UK) was used to measure both TS and EAB. Briefly, the film strip (20 mm × 60 mm) was vertically fixed onto the analyzer with two grips initially spaced 20 mm apart, and then stretched at a force rate of 2 mm/s. TS was obtained by dividing peak load by the cross-sectional area of film strip (film thickness × 20 mm). The EAB was defined as the percentage increase in distance traveled by the film strip before rupture, relative to the initial length of 20 mm.

#### 2.5.5. Optical Properties

The color of the films was measured using a colorimeter (LabScan XE, HunterLab, Reston, VA, USA) and expressed as L, a, and b values. The opacity of the films was calculated as the absorbance at 600 nm divided by the film thickness (mm).

#### 2.5.6. DPPH Free Radical Scavenging Activity

The antioxidant activity of films was determined using the DPPH method [22]. In brief, 100 mg of film was added into 5 mL of 95% ethanol, then the samples were stirred vigorously for 3 min. Next, 0.1 mL of extracted solution was added to 2.9 mL of DPPH solution (1 mM), and the mixture was kept in the dark at room temperature for 30 min. The sample containing 0.1 mL of 95% ethanol and 2.9 mL of DPPH solution was used as control. The absorbance of mixture solution was read at 515 nm and the DPPH free radical scavenging activity was expressed as follows:

(4) $$\mathrm{DPPH}\;\mathrm{inhibition}=\frac{\mathrm{Ac}-\mathrm{As}}{\mathrm{Ac}}\;\times \;100,$$

where Ac is the absorbance of the control group, and As is the absorbance of the sample.

#### 2.5.7. α-Tocopherol Release

α-Tocopherol release was measured according to Darbasi et al. [23] with some modifications. Specifically, film (2 cm × 2 cm) was placed into a glass tube with a cap and immersed in 30 mL of 95% ethanol. All samples were placed in the dark at room temperature for 4 days, during which 3 mL of the sample solutions were withdrawn from the tubes to measure the absorbance at 292 nm periodically until the absorbance was unchanged. After each measurement, the sampled solution was returned to the tube to ensure that the total amount of α-tocopherol remained constant throughout the entire procedure. The release profile was determined by calculating the percentage of free α-tocopherol in ethanol relative to the total amount of α-tocopherol present in the film, as a function of storage time. 

### 2.6. Statistical Analysis

All experiments were performed in triplicate, and the results were presented as the mean ± standard deviation (SD). To analyze the differences between mean values, an ANOVA and Duncan’s test were conducted using SPSS 25.0 software (SPSS Inc., Chicago, IL, USA) with a significance level of 0.05.

## 3. Results

### 3.1. Film Surface Morphology

**Figure 1:**
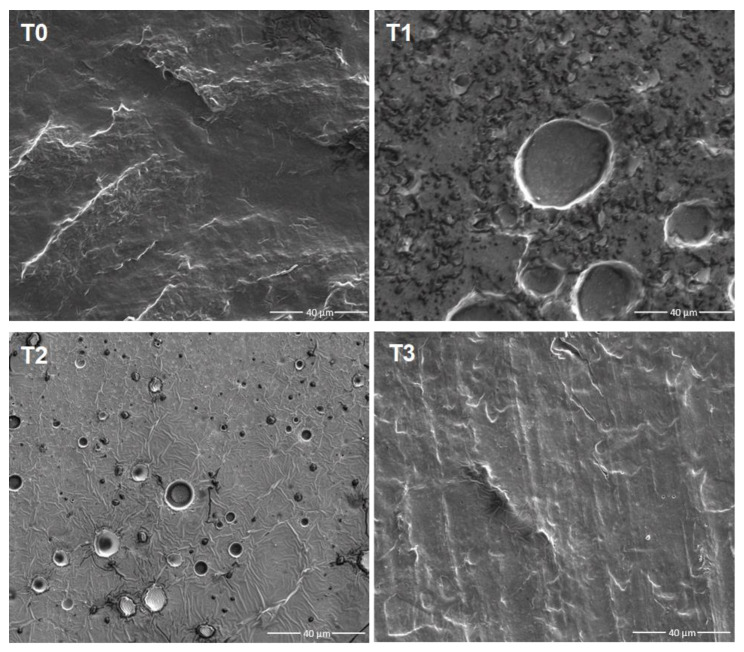
The SEM images of films. T0: PPI film (T0 is the PPI film without α-tocopherol); T1: PPI+TOC film (T1 (PPI+TOC) film is the film formed by direct addition of α-tocopherol (in ethanol) into PPI dispersion); T2: PPI_pH12_+TOC film (T2 (PPI_pH12_+TOC) film is the film formed by treating PPI dispersion with the pH-shifting process, followed by direct addition of α-tocopherol (in ethanol)); T3: PPI_pH12_+NanoTOC film (T3 (PPI_pH12_+NanoTOC) film is the film formed by treating PPI dispersion with pH-shifting process, followed by addition of α-tocopherol nanoemulsion).

The microstructure of films provides information about the arrangement of components within the films as well as their interactions [24]. To characterize the changes in microstructure that occurred with the addition of α-tocopherol, we analyzed the surface morphologies of PPI films using SEM. As shown in Figure 1, the surface of T0 was not smooth and had some bulges, which might be due to the incompletely dissolved protein particles [25]. Despite the unsmooth surface, T0 still had a tight and homogeneous structure without any pores, indicating good compatibility between the film components (e.g., PPI and glycerol). After the addition of α-tocopherol, T1 film showed a rougher surface with numerous small granules and several large holes, indicating structural discontinuities of the film. This was not surprising considering the incompatibility of hydrophobic α-tocopherol and the hydrophilic film-forming dispersions [22,25]. However, after the pH-shifting treatment, the compatibility between α-tocopherol and the film matrix increased, as supported by the reduced pore size and smoother surface of T2 film compared to T1. The pH-shifting treatment of PPI was reported to improve its solubility and surface hydrophobicity of PPI [17], which led to better emulsification of α-tocopherol, as seen in our case. Meanwhile, it was apparent that there were still small pores and cracks distributed on T2 film, which might contribute to poor mechanical properties of the film. To overcome this, α-tocopherol was incorporated into oil-in-water emulsion using PPI as the emulsifier, and then added to the pH-shifting-treated PPI dispersion for film formation, to further increase its miscibility. Indeed, T3 film displayed a more homogeneous and compact film surface compared to T1 and T2 films.

### 3.2. Solubility and Moisture Content (MC)

Solubility and MC are two properties that are commonly used to evaluate the water sensitivity of films for food packaging. The results presented in Table 2 showed that T1 film exhibited the lowest solubility compared to other films, suggesting that the hydrophobic nature of α-tocopherol contributed to T1′s water resistance. Similar results have been reported by Martins & Vicente [26] and Agudelo-Cuartas et al. [10]. However, both T2 and T3 films showed higher solubility. The increase in film solubility may be related to the partial unfolding of the PPI structure due to the increased ionic interaction under extreme alkaline conditions created by the pH-shifting process. This can result in the formation of a “molten globule” structure, which has been shown to substantially increase protein solubility in previous studies [17,27]. The improved protein solubility may have promoted more soluble proteins to leach out of the films, which further increased their solubility. 

There was no significant difference in MC among all samples, indicating that the addition of α-tocopherol and the pH-shifting process did not result in any significant alteration of the hygroscopicity of the PPI films. It may be due to the relative low level of α-tocopherol (0.5% of total film-forming dispersion) incorporated in the film, which may be insufficient to significantly influence the MC [28].

### 3.3. Water Vapor Permeability (WVP)

WVP is another important property for biodegradable films, as water vapor can accelerate food deterioration and spoilage. A lower WVP value is generally desirable to prevent water molecules from coming into contact with food. Table 2 indicated no significant variation in WVP values for all samples, revealing that the inclusion of α-tocopherol had no impact on the WVP characteristic of PPI film. Also, pH-shifting and addition of α-tocopherol nanoemulsion addition did not enhance the films’ water vapor barrier property. These findings contradicted with some prior studies that suggested that adding hydrophobic compounds improved water barrier properties [25,29]. However, it should be noted that some other studies indicated that adding α-tocopherol actually raised WVP values of edible films [10,12,26]. In fact, adding α-tocopherol can exert various effects on the WVP value of a film. For example, it can increase film hydrophobicity, thereby reducing WVP values, but it can also disrupt the film structure (supported by SEM results), which potentially increases WVP values by facilitating water molecules passing through the film matrix. In addition, the film’s thickness, hydrophilic-hydrophobic ratio, and polymeric chain mobility also influence the WVP value [30]. In this study, the lack of significant difference may be a net result of multiple factors.

### 3.4. Tensile Strength (TS) and Elongation at Break (EAB)

TS and EAB are important indicators to characterize the mechanical properties of edible films. TS refers to the maximum stress that a film can withstand before breaking, and EAB indicates the amount of deformation a film can undergo before rupturing. The effects of α-tocopherol and pH-shifting treatments on TS and EAB of PPI film were shown in Table 2. Compared to T0, direct addition of tocopherol (T1) displayed a reduction in both TS and EAB. This was not surprising because the addition of hydrophobic substances to the aqueous film-forming dispersion can inevitably lead to an incontinuous film structure (as evidenced by Figure 1), thereby reducing the mechanical properties of the film [25]. However, pH-shifting treatment significantly improved the mechanical properties of T2 film compared to T1, indicating its potential for facilitating the incorporation of hydrophobic active compounds, such as α-tocopherol, into the film matrix. This finding is important as it has not been reported in previous literature. The improved mechanical properties of T2 film induced by pH-shifting treatment may be explained by two reasons. Firstly, pH-shifting treatment can improve the emulsifying ability of PPI [15,17,27], which can lead to better incorporation of α-tocopherol into PPI film (as supported by SEM results). Secondly, the alkaline pH treatment unfolded the structure of protein and produced more soluble protein aggregates from dissociated protein subunits, which enhanced the interactive film network and increased the elasticity of the protein film [31]. In the meantime, the mechanical properties of T2 are still not as good as T0. To further improve this, α-tocopherol was mixed into the film in the form of a nanoemulsion (T3 film), and the results showed significant increase of both TS and EAB as compared to T1 or T2. Moreover, the EAB of T3 film even surpassed that of T0. In another study, it was also reported that adding α-tocopherol nanoemulsion led to the increase of elongation at break of whey protein film from 29.4% to 48.6% [10]. In fact, there have been several studies indicating that incorporating hydrophobic compounds into edible films in the form of nanoemulsions can significantly improve their elongation properties [8,32,33]. This phenomenon has been attributed to the plasticizing role of hydrophobic compounds in the film matrix [8]. In addition, the smaller size of oil droplets induced by nanoemulsion can increase the heterogeneity of biopolymer network, thus decreasing chain-chain interaction and increasing the plasticizing effect [34]. These findings demonstrated that pH-shifting treatment, together with α-tocopherol nanoemulsion effectively overcame the damage of α-tocopherol to the mechanical properties of PPI film.

### 3.5. Optical Properties

Color and opacity of edible film are important because they can have a significant impact on the appearance, shelf life, and consumer perception of the packaged product. Table 3 showed that the *L* value of T1 film increased as compared to T0 while the *a* and *b* values decreased, suggesting that the incorporation of α-tocopherol resulted in a film with a whiter appearance and lower levels of yellow and red hues. This result is inconsistent with previous studies, which reported that the addition of α-tocopherol could increase the yellowish hue of whey protein film [10] and polylactic acid film [11]. This is because whey protein and polylactic acid protein films have a white color, so the addition of yellow-colored α-tocopherol is expected to increase the yellowish hue of the films. However, the color of PPI film is already yellowish compared to that of α-tocopherol, so the addition of α-tocopherol did not increase the yellow color of PPI film but decreased it instead. Compared to T1 film, both T2 and T3 films showed a significant decrease in *L* value and a significant increase in *b* value. The results above suggest that the addition of α-tocopherol can significantly alter the color of PPI film, and pH-shifting and nanoemulsion addition also contributed to changes in color. Regarding the opacity, all samples showed significant differences when compared to each other. Specifically, T0 had the highest opacity (2.72) while T2 film had the lowest (0.92). In a preliminary experiment, we found that pH-shifting treatment could significantly reduce the opacity of PPI film, which could be the reason that T2 film showed a much more transparent appearance than T0 and T1 films. A similar result was reported in a previous study, which found that pH_12_-treated SPI could form a transparent film [31]. It may be related to the improved solubility and decreased particle size of protein molecules induced by pH-shifting treatment [27], which would allow more soluble protein particles with smaller size into the film formation and less undissolved particles distributed on the surface upon drying, thus, producing a homogeneous and transparent film. However, the addition of α-tocopherol in the nanoemulsion form led to a significant increase in the opacity of T3 film as compared to T2. This might be explained by the presence of microscale particles in the polymer matrix that can scatter light, resulting in a reduction in light transmittance and optical clarity [35].

### 3.6. Antioxidant Activity

**Figure 2:**
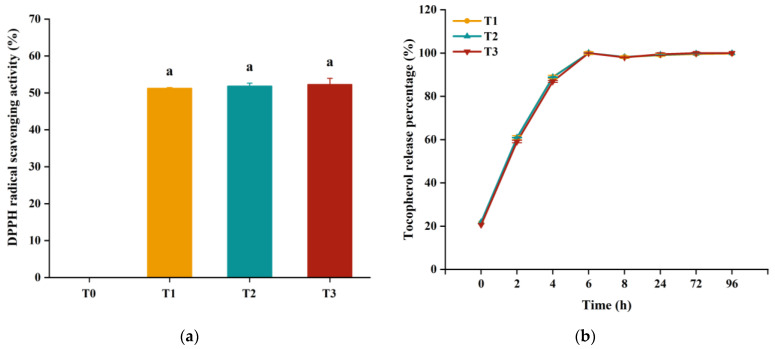
(**a**) The DPPH scavenging activity of films. (**b**) The release rate of α-tocopherol in films. T0: PPI film (T0 is the PPI film without α-tocopherol); T1: PPI+TOC film (T1 (PPI+TOC) film is the film formed by direct addition of α-tocopherol (in ethanol) into PPI dispersion); T2: PPI_pH12_+TOC film (T2 (PPI_pH12_+TOC) film is the film formed by treating PPI dispersion with the pH-shifting process, followed by direct addition of α-tocopherol (in ethanol)); T3: PPI_pH12_+NanoTOC film (T3 (PPI_pH12_+NanoTOC) film is the film formed by treating PPI dispersion with pH-shifting process, followed by addition of α-tocopherol nanoemulsion). The lowercase letters “a” in Figure 2a indicate that there was no statistically significant difference among films with different treatments.

The main objective of incorporating α-tocopherol into edible films is to enhance their antioxidant activity, which can help to inhibit the oxidation of food product. The DPPH radical scavenging capacity and the release rate of α-tocopherol were measured to evaluate the antioxidant activity of PPI films. As shown in Figure 2a, all PPI films containing α-tocopherol displayed DPPH scavenging activity with no significant differences among them. This result was in accordance with previous studies, which reported that the addition of α-tocopherol could enhance the DPPH radical scavenging activity of edible films [7,8,10]. Regarding the release rate of α-tocopherol (shown in Figure 2b), all three films exhibited a similar release rate (*p* > 0.05), with a burst release occurring within the first 6 h. After 6 h, the release rate remained consistent, indicating that all α-tocopherol was released from the films into the ethanol phase. The release rate is slower than that of chitosan film, where burst release was observed during the initial period of the experiment (less than 1 h) [23], indicating extended release of α-tocopherol from the PPI film. The release rate of antioxidant compounds from edible film is determined by various factors, including polymer composition, molecular mass and polydispersity, particle size of the matrix, and the simulant environment [11,36]. Therefore, it is not surprising that the release rate is different in various edible films. These results demonstrate that the addition of α-tocopherol successfully enhanced the antioxidant activity of the PPI film. Furthermore, neither pH-shifting treatment nor α-tocopherol addition methods (direct addition vs. nanoemulsion addition) affected the film’s antioxidant activity.

## 4. Conclusions

In this study, α-tocopherol was successfully incorporated into PPI film without sacrificing its mechanical properties. Specifically, direct addition of α-tocopherol led to a rough and discontinuous film with large holes, resulting in a significant decrease in TS and EAB properties of PPI film. In contrast, addition of α-tocopherol after pH-shifting treatment of PPI resulted in a smoother surface with smaller pores, significantly improved TS and EAB values, and increased the transparency of PPI film. Building upon this, addition of α-tocopherol in the form of a nanoemulsion further modified the surface morphology and considerably improved the elongation property. In addition, the inclusion of α-tocopherol and pH-shifting process significantly affected the color and transparency of PPI film, but had little effect on the solubility, MC, and WVP properties. All PPI films containing α-tocopherol had similar release rates and DPPH scavenging abilities. In summary, the combination of pH-shifting and addition of α-tocopherol in the nanoemulsion form is a promising method for developing antioxidant protein-based films with desirable mechanical properties for food packaging applications such as wrapping meats, fruits, and vegetables. 

## Figures and Tables

**Table 1:** Formulation of PPI film-forming dispersions loaded with α-tocopherol based on weight percentage.

Samples	PPI (%)	Distilled Water (%)	α-Tocopherol		Glycerol (%)
			Direct Addition (%)	Nanoemulsion Addition (%)	
T0 (PPI)	7.5	88.75	0	/	3.75
T1 (PPI+TOC)	7.5	88.25	0.5	/	3.75
T2 (PPI_pH12_+TOC)	7.5_pH12_	88.25	0.5	/	3.75
T3 (PPI_pH12_+NanoTOC)	7.0_pH12_	59.25	/	30 *	3.75

PPI: pea protein isolate; TOC: α-tocopherol; PPI_pH12_: pea protein isolate treated with pH-shifting process; NanoTOC: α-tocopherol nanoemulsion. * 30% nanoemulsion contains 0.5% α-tocopherol and 0.5% PPI in the final dispersion.

**Table 2:** The solubility, moisture content (MC), water vapor permeability (WVP), tensile strength (TS), and elongation at break (EAB) of films. T0: PPI film (T0 is the PPI film without α-tocopherol); T1: PPI+TOC film (T1 (PPI+TOC) film is the film formed by direct addition of α-tocopherol (in ethanol) into PPI dispersion); T2: PPI_pH12_+TOC film (T2 (PPI_pH12_+TOC) film is the film formed by treating PPI dispersion with the pH-shifting process, followed by direct addition of α-tocopherol (in ethanol)); T3: PPI_pH12_+NanoTOC film (T3 (PPI_pH12_+NanoTOC) film is the film formed by treating PPI dispersion with pH-shifting process, followed by addition of α-tocopherol nanoemulsion). Different lowercase letters in the same column indicate statistically significant differences among films with different treatments.

Samples	Solubility (%)	Moisture Content (%)	WVP (g mm h^−1^ m^−2^ kPa^−1^)	Tensile Strength (MPa)	Elongation at Break (%)
T0	62.34 ± 4.00 ^b^	19.74 ± 2.52 ^a^	2.56 ± 0.20 ^a^	1.56 ± 0.18 ^c^	124.87 ± 7.26 ^b^
T1	50.97 ± 7.45 ^a^	16.18 ± 6.48 ^a^	2.36 ± 0.07 ^a^	0.70 ± 0.01 ^a^	32.96 ± 9.98 ^a^
T2	58.63 ± 0.52 ^ab^	19.22 ± 0.80 ^a^	2.41 ± 0.00 ^a^	1.07 ± 0.11 ^b^	107.70 ± 9.31 ^b^
T3	59.80 ± 1.45 ^b^	20.86 ± 1.21 ^a^	2.32 ± 0.14 ^a^	1.52 ± 0.01 ^c^	198.54 ± 55.24 ^c^

**Table 3:** The *L*, *a*, *b* values and opacity of films. T0: PPI film (T0 is the PPI film without α-tocopherol); T1: PPI+TOC film (T1 (PPI+TOC) film is the film formed by direct addition of α-tocopherol (in ethanol) into PPI dispersion); T2: PPI_pH12_+TOC film (T2 (PPI_pH12_+TOC) film is the film formed by treating PPI dispersion with the pH-shifting process, followed by direct addition of α-tocopherol (in ethanol)); T3: PPI_pH12_+NanoTOC film (T3 (PPI_pH12_+NanoTOC) film is the film formed by treating PPI dispersion with pH-shifting process, followed by addition of α-tocopherol nanoemulsion). Different lowercase letters in the same column indicate statistically significant differences among films with different treatments.

Samples	*L*	*a*	*b*	Opacity
T0	84.08 ± 0.27 ^b^	2.86 ± 0.07 ^b^	18.57 ± 0.47 ^b^	2.72 ± 0.01 ^d^
T1	85.10 ± 0.43 ^c^	2.30 ± 0.09 ^a^	14.44 ± 1.10 ^a^	1.96 ± 0.00 ^c^
T2	84.02 ± 0.91 ^b^	2.36 ± 0.20 ^a^	17.06 ± 1.85 ^b^	0.92 ± 0.04 ^a^
T3	81.73 ± 1.01 ^a^	3.02 ± 0.53 ^b^	21.46 ± 2.76 ^c^	1.81 ± 0.01 ^b^

## Data Availability

The data presented in this study are available in article.
